# The implementation of a smart safety protection system using DL and IoT technologies

**DOI:** 10.1371/journal.pone.0354273

**Published:** 2026-09-08

**Authors:** Kaouther Omri, Hanen Himdi, Manal M. Khayyat, Ala’ A. Eshmawi, Mashael M. Khayyat

**Affiliations:** 1 Department of Computer and Network Engineering, College of Computer Science and Engineering, University of Jeddah, Jeddah, Saudi Arabia; 2 Department of Computer Science and Artificial Intelligence, College of Computer Science and Engineering, University of Jeddah, Jeddah, Saudi Arabia; 3 Department of Computer Science and Artificial Intelligence, College of Computing, Umm Al-Qura University, Makkah, Saudi Arabia; 4 Department of Cybersecurity, College of Computer Science and Engineering, University of Jeddah, Jeddah, Saudi Arabia; 5 Department of Information Systems and Technology, College of Computer Science and Engineering, University of Jeddah, Jeddah, Saudi Arabia; Maulana Abul Kalam Azad University of Technology West Bengal, INDIA

## Abstract

Safety has always been a concern. Thus, employing and utilizing technology is vital to help society address this concern. This paper looks at the design and implementation of a Smart Safety Protection System (SSPS), which employs the Internet of Things (IoT) to solve the issue of real-time monitoring, geolocation tracking, and emergency alert features. The proposed system is a wearable device that uses sensors and GPS to send continuous data to both an application on smartphones and a database on the cloud. The system’s features include detecting unauthorized removal of the device as well as falls and abnormal temperature or heart rate. If anything happens as mentioned, the system will send alerts to the linked mobile application. A Deep Learning (DL) model is integrated into the SSPS system using multimodal WESAD sensor data. A trained Multi-Layer Perceptron (MLP) neural network achieved 93% accuracy when optimized with Adam and Softmax, enabling reliable identification of Monitored Individuals’ emotional states. Steps of functional and integration testing of the prototype proved the reliability of sensor accuracy, data transmission speed, and real-time emergency response. Ultimately, this work emphasizes the potential of using DL and the IoT to improve Monitored Individuals’ safety.

## Introduction

Safety, in general, is a concern that always draws researchers’ attention. Monitored Individuals may encounter several types of dangers, intentional such as kidnapping, and unintentional such as accidental injuries. Some statistics show that over 900,000 Monitored Individuals die worldwide every year from injuries that can be prevented. It is assumed that if caregivers can give timely intervention or have proper awareness, most of these deaths can be avoided [1]. Recent studies in Saudi Arabia have shown a need for tools that can assist in protecting Monitored Individuals, especially in schools, crowded areas, and urban neighborhoods [2]. The IoT arena provides promising tools to bridge this gap in safety. Using IoT has proven its ability to respond to potential risks immediately [3]. Unfortunately, the available solutions focus on basic things like tracking using GPS or alerts using SMS [4]. Furthermore, many devices mostly lack secure biometric authentication as well as real-time health checks or methods to prevent tampering and theft. This work aims to develop a system that combines all available protection features into several small sensors to protect Monitored Individuals by means of a device that tracks their actual location, the rate of falling shocks, biometrics, and the temperature of the Monitored Individual, to build a reliable surveillance and communication channel between the Monitored Individual and the family. The main objectives of this work are to develop an easy-to-use safety device and application that enables families to track their members inside or outside the house; to facilitate the survival process by sending an emergency warning in case of injury; to provide health and safety services that could save the Monitored Individual’s life in case of an attack, including heart rate, temperature, and actual location; to allow secure sharing of the Monitored Individual’s data; to warn and inform the Caregiver if the Monitored Individual has reached a place he or she is not supposed to be in; and lastly, to provide a strong lock that could not be opened without the Caregiver, by applying biometric authentication.

The motivation for this research stems from the urgent need to enhance Monitored Individuals’ safety using modern wearable technology. In many situations, Monitored Individuals may go missing in crowded environments or find themselves in unsafe conditions without adult supervision, which can lead to serious harm or abduction. To address these risks, we propose the development of a specialized application integrated with a wearable sensor-based bracelet designed to ensure continuous monitoring and rapid response. The system incorporates biometric sensors, a real-time GPS tracking feature, and an intelligent warning mechanism capable of detecting potential incidents that could endanger the Monitored Individual. To strengthen the Smart Safety Protection System’s (SSPS) analytical capabilities, a deep learning classifier was incorporated to interpret physiological signals and detect emotional states. Using WESAD data and a trained MLP model, the system accurately distinguishes between various emotional states. This integration transforms the SSPS from a simple sensing device into an intelligent, context-aware safety tool.

The rest of this paper is organized as follows. First, a summary of various studies related to protection and safety is presented, highlighting the strengths and limitations of each approach. Next, the implementation of the proposed Smart Safety Protection System is described in detail, including its main components and operational process. Then, the integration of Deep Learning (DL) into the SSPS system is developed and examined. Finally, the paper discusses the results, provides recommendations, and outlines directions for future research. The proposed system aims to offer innovative features that assist in ensuring the safety and well-being of Monitored Individuals.

## Literature review

The authors in [5] conducted a review of IoT wearables in Monitored Individuals’ health to understand how these wearables support real-time monitoring of their vitals. In their systematic mapping approach, after screening over 2000 articles, their analysis highlighted that most research focused on elderly care, such as fall detection or prevention in older Monitored Individuals. Hence, there is a noticeable gap in the use of IoT devices for Monitored Individual-specific applications. In the review, temperature, heart rate, motion, and Global Positioning System (GPS) are the widely used/preferred IoT features, which result in enhancing Monitored Individual safety and well-being. However, it also comes with additional concerns regarding privacy, comfort, and data security. Overall, the review concludes that IoT wearables offer great potential for Monitored Individual tracking and healthcare, but this field remains largely unexplored.

Another review performed by Saeedbakhsh et al. [6] also explores the use of IoT to enhance Monitored Individual care and safety. Overall, the authors analyzed 273 papers across well-known databases, such as PubMed and Web of Science, of which they evaluated only 29 papers based on their inclusion criteria. Their first observation was that most of the IoT-based Monitored Individual safety systems were developed in India, Italy, and China for indoor environments like homes and hospitals. Hence, many studies lack coverage of outdoor environments. Similar to [5], this review also highlighted that temperature, heart rate, and motion sensors are widely used for Monitored Individual care applications. However, considering the limited battery life, such devices are often combined with cloud computing for real-time monitoring. Lastly, the review highlighted that IoT has great potential for Monitored Individual care and well-being, but future research should explore innovative applications like smart toys and intelligent car seats.

A conceptual design of a smart band to remotely monitor the Monitored Individual’s safety and health conditions is presented in [7]. The design of their smart band consists of various sensors, including heart rate, temperature, respiration, sleep quality, GPS, and motion detection. Furthermore, it incorporates an emergency button for alerts and screen recording. Considering the low energy of the smart band, the authors proposed using the cloud for secure data processing from sensors, which can later be visualized on a mobile app. Lastly, the authors conducted an online survey and semi-structured interviews with 50 Caregivers to evaluate its practicality. The responses show strong support for this concept, particularly for its real-time updates and alerts.

Nirag et al. [8] also designed an IoT-based wearable device to enhance Monitored Individual monitoring. Their proposed device includes features like emergency communication and real-time location tracking to improve overall Monitored Individual safety. The device consists of several sensors, including heart rate, temperature, and an accelerometer, to detect panic or abnormal conditions. It also integrates a GPS for positioning and for sending alerts via SMS to Caregivers and nearby police stations when a threat or accident is detected. The system hardware was implemented using NodeMCU, Arduino IDE, and Android Studio, which makes it affordable and easy to use. Overall, the prototype proved successful in real-time tracking and automatic alerts to locate missing Monitored Individuals and notify emergency contacts.

Another IoT-based wearable system, mainly a prototype, to locate and monitor Monitored Individuals is presented in [9]. The main advantage of this device over existing systems [6,7] is the use of GPS and the Global System for Mobile Communications (GSM). As a result, it can be used with any cell phone, without necessarily requiring an expensive smartphone. The proposed system uses several sensors (temperature, ultrasonic, infrared, accelerometer) connected to an Arduino board, along with a Raspberry Pi and camera for live video streaming. When a Monitored Individual presses the emergency button or leaves a safe zone, the system sends an SMS alert to the Caregivers with the Monitored Individual’s GPS location and a Google Maps link. Furthermore, it allows Caregivers to view a live video feed through a web or mobile app.

An IoT-based transport safety mechanism is presented in [10] to enhance the safety of school members traveling by bus in Oman. The proposed system consists of Radio Frequency Identification (RFID), Infrared (IR), and MQ3 alcohol sensors to monitor boarding activity, student attendance, and driver behaviour/activity. The key idea behind the proposed mechanism is that each Monitored Individual carries an RFID card, and when scanned upon entering or leaving the bus, the system records attendance and sends notifications to Caregivers and school administrators. In contrast, the IR sensors ensure that no Monitored Individual remains inside the bus, while the MQ3 alcohol sensor detects unsafe driver conditions. All sensor data are processed through a NodeMCU microcontroller and transmitted via Wi-Fi to a cloud server, with real-time updates accessible through a mobile application using Google Maps.

Sundarajoo et al. [11] developed a fully functional prototype of a remote baby surveillance system that integrates RFID and GPS technologies to enhance Monitored Individual safety and comfort. Their proposed system consists of microphones, IRs, motion, moisture, and temperature sensors to detect various parameters/activities, such as a baby’s crying, movement, sleep, and environmental conditions. The prototype also consists of an Arduino Uno microcontroller that collects and processes this data and transmits it via Wi-Fi to a mobile application. RFID is used for indoor tracking, while GPS tracks the outdoor location of the baby. Additional features include an IP camera for live monitoring and white noise to soothe the baby. In addition to electrical energy, it can also be powered by solar, making it a sustainable solution.

Yashwanth et al. [12] present an Artificial Intelligence (AI) and edge-based real-time Monitored Individual abduction detection and alert system. Their proposed system uses a multi-agent framework where each agent incorporates Vision-Language Models (VLMs) deployed on a Raspberry Pi. While most IoT devices use cloud processing, the video is processed in the proposed system locally using the Pi’s camera. The key idea behind this is to improve the speed and privacy of the data. Furthermore, it integrates an image and situation analyzer (both AI agents) to examine video feeds and detect suspicious actions. In such cases, it automatically sends an SMS or WhatsApp message with images and event details through the Twilio API. Experimental testing demonstrated 90% accuracy with an average response time of seven seconds, making it a practical solution for Monitored Individual safety monitoring.

The authors in [13] investigated the security and privacy risks of internet-connected children’s toys. In their analysis, they examine three commercially available products: a hydration tracker, a smart pet, and a fitness band. The authors performed various network monitoring and security testing approaches, where they found several hidden vulnerabilities that violated privacy policies and the Children’s Online Privacy Protection Act (COPPA) regulations. A few major issues include unencrypted data transfers, weak authentication, access to deleted photos, and sending Monitored Individual data to third parties. The key message of this study is that many toy makers ignored basic protections like encryption, which could lead to data theft or tracking of Monitored Individuals.

A context-aware IoT-based access control system, mySafeHome, is presented in [14] to protect Monitored Individuals from harmful online content through dynamic supervision. The proposed system utilizes family dynamics to determine and provide safe internet access for Monitored Individuals. mySafeHome consists of a Raspberry Pi that works as a smart router connected to OpenHAB and smart home devices. It measures the distance between a Caregiver’s and a Monitored Individual’s device through Received Signal Strength Indicator (RSSI) and uses machine learning to improve accuracy. Overall, mySafeHome automatically adjusts internet access based on Caregiver proximity and calendar data, helping improve Monitored Individuals’ online safety in smart homes.

The authors in [15] present another IoT-based system for real-time Monitored Individual safety monitoring. The proposed system integrates multiple sensors (LM35 and PLSNSR1) and communication modules into a Raspberry Pi to track a Monitored Individual’s body temperature and pulse rate. It consists of a voice recognition module that listens for specific/help-related keywords, such as “Help Me” or “Save Me,” which trigger automatic image capture via a Pi camera. After that, the module sends email and SMS alerts to Caregivers or local authorities using Twilio. The IoT system also provides the feature of live video streaming via YouTube to track real-time events remotely. This work demonstrates how combining IoT sensing, speech, and multimedia transmission can significantly enhance Monitored Individual safety and emergency response.

Similarly, Chandnani et al. [16] introduced an AI-based smart cradle to enhance infant safety and comfort through real-time monitoring. The cradle consists of numerous sensors for temperature, humidity, gas, noise, and crying, along with motion and weight detection to track a baby’s environment and posture continuously. The system is mainly built using Raspberry Pi and NodeMCU controllers. The proposed system uses machine learning to analyze sensor data and automatically trigger responses based on the conditions, including temperature control, air filtering, or rocking the cradle. Furthermore, a mobile app provides Caregivers with live updates, remote control, and data visualization. Another promising feature of the system is the integration of AES-256 encryption and blockchain-inspired data protection to enhance system security. The prototype achieves more than 90% accuracy for all tasks, highlighting how IoT and AI can work together to enhance Monitored Individual monitoring and protection.

Along the same line, physiological stress detection has become an active research area, supported by the growing availability of multimodal datasets and advancements in machine and DL techniques. Several review studies have examined the landscape of stress monitoring to highlight methodological trends and existing gaps.

A comprehensive survey by Halkiopoulos et al. [17] integrates neuroimaging and deep learning to enhance emotion detection. It concludes that combining techniques such as fMRI, EEG, and MEG with models like Convolutional Neural Networks (CNNs) and Generative Adversarial Networks (GANs) improves classification accuracy and interpretability. However, challenges persist in data availability, model transparency, and ethical concerns. The authors call for unified, ethical frameworks that merge cognitive neuroscience with algorithmic innovation.

Similarly, the review by Samal et al. [18] highlights deep learning’s superiority over traditional machine learning methods in recognizing emotional states. It reviews key datasets, feature extraction methods, and classifiers, emphasizing multimodal and hybrid models for real-time, adaptive emotion recognition. The study points to future work in feature optimization and model generalization for broader applications.

A third review by Ma et al. [19] provides a structured taxonomy of EEG-based emotion recognition, distinguishing between subject-dependent and subject-independent models. It synthesizes recent advances in preprocessing, modeling, and dataset development, underscoring EEG’s reliability compared to facial or vocal cues. The authors recommend standardized datasets and explainable AI for improving reproducibility and interpretability.

Collectively, these review articles underscore the need for robust benchmark datasets such as WESAD and emphasize the importance of developing models that balance accuracy, interpretability, and deployability. The following studies illustrate how different researchers have applied deep learning techniques to the WESAD dataset, also employed in the present work, for stress recognition.

Chakraborty et al. [20] developed a lightweight CNN–LSTM model trained on wrist-based PPG signals, achieving near-97% accuracy in binary stress detection. Their work is distinguished by demonstrating successful deployment on embedded hardware, supporting real-time inference for wearable devices. While practical and computationally efficient, their approach remains limited to two emotional states and has not been validated under noisy, real-world conditions.

In a related study, Mzoughi et al. [21] advance stress-recognition methodology by proposing an attention-driven hybrid CNN–BiLSTM architecture using ECG signals. The model achieves approximately 95–96% accuracy across multiple emotional classes and benefits from an attention mechanism that highlights salient temporal regions contributing to classification. An important contribution of their work is the adversarial-robustness analysis, which reveals resilience to several gradient-based attacks. However, the architecture is computationally demanding, and robustness is assessed only under synthetic attack scenarios, leaving open questions regarding environmental noise and device-level variability.

Similarly, Nazarova et al. [22] combine feature engineering and deep learning by first selecting an optimal subset of handcrafted ECG/HRV features using a Random Forest ranking procedure, then feeding these features into a CNN–LSTM framework. Their method achieves around 97% accuracy in distinguishing stress, amusement, and baseline states, while offering improved computational efficiency and some interpretability due to the reduced feature set. Nevertheless, this approach relies on the quality of engineered features and, like the others, has been validated only in controlled experimental settings rather than naturalistic environments.

Across both the review literature and the empirical studies, there is strong evidence that deep-learning approaches, especially hybrid CNN–RNN models, are highly effective for physiological stress recognition. Studies leveraging the WESAD dataset consistently report accuracies between 95% and 97%, demonstrating that physiological markers such as PPG and ECG contain rich, learnable patterns indicative of stress. Review works emphasize, however, that high performance on controlled datasets does not guarantee real-world robustness, as issues such as motion artifacts, sensor variability, demographic diversity, and environmental noise remain insufficiently addressed. Collectively, the literature indicates that while current models achieve excellent performance on benchmark datasets like WESAD, which the present work also uses, there is a critical need for approaches that generalize better to unconstrained settings, integrate robustness mechanisms, and remain computationally efficient for wearable deployment.

To provide a concise comparison of existing studies, Table 1 summarizes their sensing inputs, key contributions, and limitations. While most of the existing works address specific aspects of safety monitoring, many are constrained by limited deployment scope, scalability, and real-world validation. Furthermore, these prior studies often lack integrated intelligent analysis. These gaps motivate the development of the proposed SSPS, which combines real-time sensing, emergency response, and intelligent analysis within a single integrated framework.

**Table 1:** Qualitative comparison of representative existing studies and their limitations.

Ref.	Sensors/Inputs	Real-Time	Key Contribution	Limitations
[5]	A review paper not a proposed work so no qualitative comparison			
[6]	A review paper not a proposed work so no qualitative comparison			
[7]	GPS, vital sensors, emergency button, camera/video input	Yes	IoT smart band enabling real-time child monitoring with geofencing and emergency SOS alerts.	Prototype-level system with limited details, reliant on connectivity, and raises privacy concerns.
[8]	GPS, GSM module, pulse sensor, temperature sensor, accelerometer, flex sensor	Yes	Wearable IoT device for real-time tracking and health monitoring with SMS alerts.	Relies on GSM, has limited indoor accuracy, and offers basic alerts with privacy concerns.
[9]	GPS/location tracking, environmental sensing, wearable device inputs	Yes	Low-cost Arduino–Raspberry Pi wearable for tracking, compatible with basic mobile phones.	Limited sensing, mainly location-based, network-dependent, and lacks advanced analytics.
[10]	RFID, IR sensors, MQ3 sensor, GPS, mobile app inputs	Yes	IoT smart bus system for student tracking, safety, and parent alerts.	Bus-only scope, network-dependent, limited automation, and potential hardware costs.
[11]	RFID, GPS, temperature sensor, wetness sensor, sound, motion/object detection sensors	Yes	IoT baby monitoring with sensors, alerts, and comfort features.	Prototype IoT system with reliability, privacy, and connectivity issues.
[12]	Camera, edge device, AI vision models (VLMs for behavior detection)	Yes	Edge AI vision system detecting suspicious interactions and sending instant alerts.	Camera-based system with false alarms, privacy risks, and high computation requirements.
[13]	Analysis paper should not be included in the qualitative comparison			
[14]	RSSI, device presence/proximity data, smart home IoT devices, contextual data	Yes	Context-aware IoT access control adapting permissions based on family proximity for child online safety.	No physical tracking; smart-home dependent with RSSI accuracy issues and prototype limitations.
[15]	A review paper not a proposed work so no qualitative comparison			
[16]	Motion sensors, sound, temperature & humidity sensors, load/pressure sensors, possibly camera + IoT connectivity	Yes	IoT–ML baby cradle monitoring and automatically adjusting based on baby conditions.	Prototype ML system with limited validation, sensor dependence, and false alarms.
[20]	PPG (photoplethysmography) physiological signals (from WESAD dataset), derived features like heart rate variability	No	Deep learning stress detection using physiological signals.	Dataset-based with no real IoT deployment or hardware, limited generalization.
[21]	ECG (electrocardiogram) physiological signals, extracted features (heart rate variability, rhythm patterns)	No	Attention-based CNN–RNN model for ECG stress detection with improved accuracy.	Dataset-based, no IoT deployment, needs ECG sensors, limited real-world testing.
[22]	ECG (electrocardiogram) signals, time-series physiological features (heart rate variability, signal morphology)	No	CNN–LSTM model for ECG stress detection using spatial and temporal features.	Dataset-based ECG model with no deployment and limited real-world generalization.

## Architecture and development of the smart safety protection system

This section presents the complete architecture of the proposed SSPS. It is organized into subsections covering the hardware construction, software development, and deep learning integration. Together, these elements form an IoT-enabled and deep learning framework for real-time state monitoring and emergency response.

### Hardware construction

To construct a functional prototype of the IoT-enabled SSPS, several hardware components were selected to provide accurate physiological monitoring, environmental sensing, and reliable connectivity. Table 2 summarizes the main hardware used in the system.

**Table 2:** Hardware and sensor components.

Component	Function	Purpose in SSPS
ESP32 microcontroller	Wi-Fi and Bluetooth connectivity	Main controller; transmits sensor data to the cloud database and mobile application
MLX90614 infrared sensor	Contactless temperature sensing	Accurately measures body temperature
MAX30100 pulse sensor	Pulse oximetry and heart rate monitoring	Captures heart rate and blood oxygen (SpO_2_) levels
MPU6050 accelerometer/gyroscope	Fall and sudden movement detection	Detects shocks, falls, and abnormal movements
A9G GPS module	Real-time GPS tracking	Provides geolocation information for the Monitored Individual
LED indicator	Visual alert	Indicates emergency or application-triggered events
Buzzer	Audible alert	Activates during SOS or emergency situations
Jumper wires	Circuit connections	Provide physical wiring between components

These components were selected to create a compact, low-power wearable device capable of capturing physiological data, movement patterns, GPS coordinates, and emergency states.

**Fig 1:**
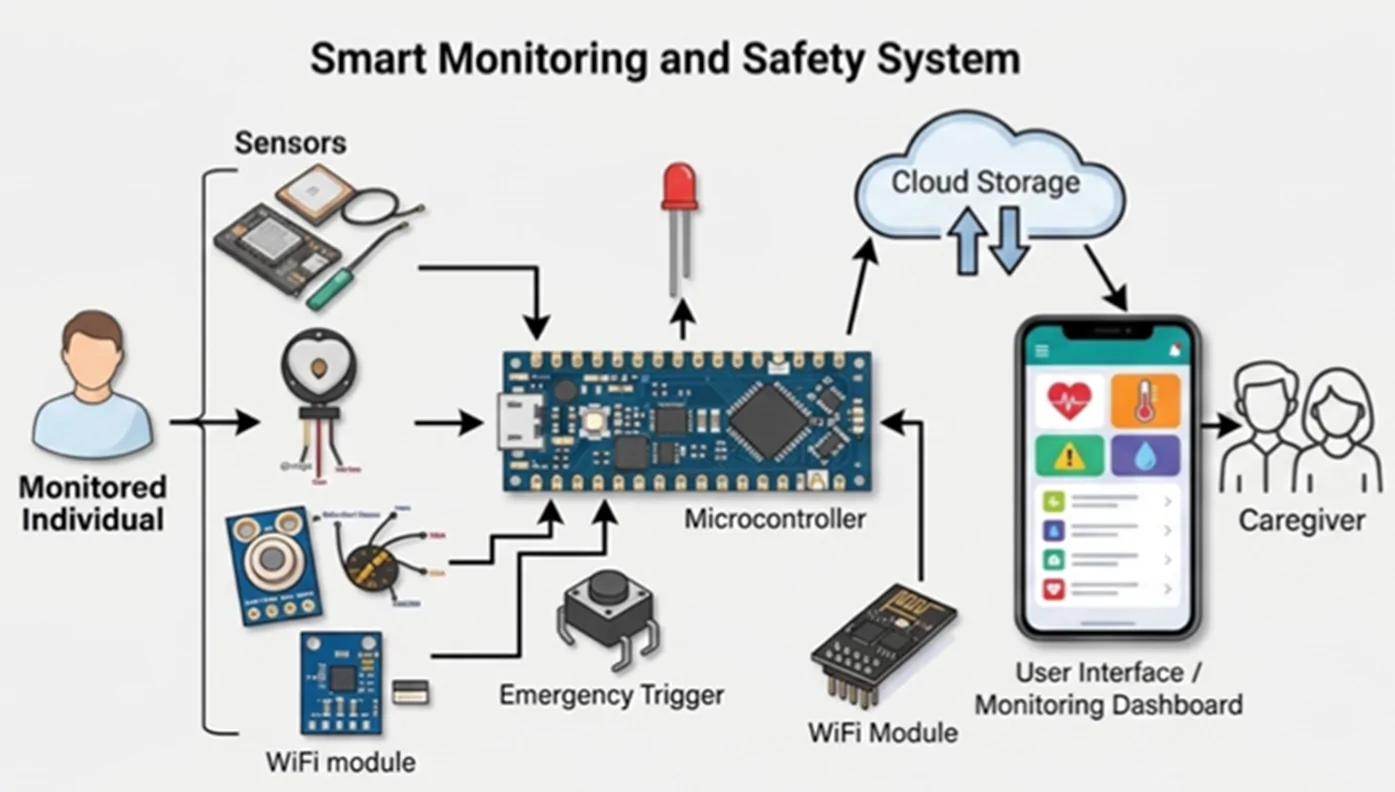
Overall system architecture.

The proposed SSPS brings together all the hardware, cloud infrastructure, and mobile applications to provide a real-time Monitored Individual safety monitoring system. The architecture involves three core components: the wearable device, the mobile application, and the cloud-based database. The wearable device is a compact, child-friendly unit equipped with sensors for body temperature, heart rate, fall detection, and GPS for real-time location tracking. The overall system interaction is presented in Fig 1, which outlines the main components and the communication flow among them.

**Fig 2:**
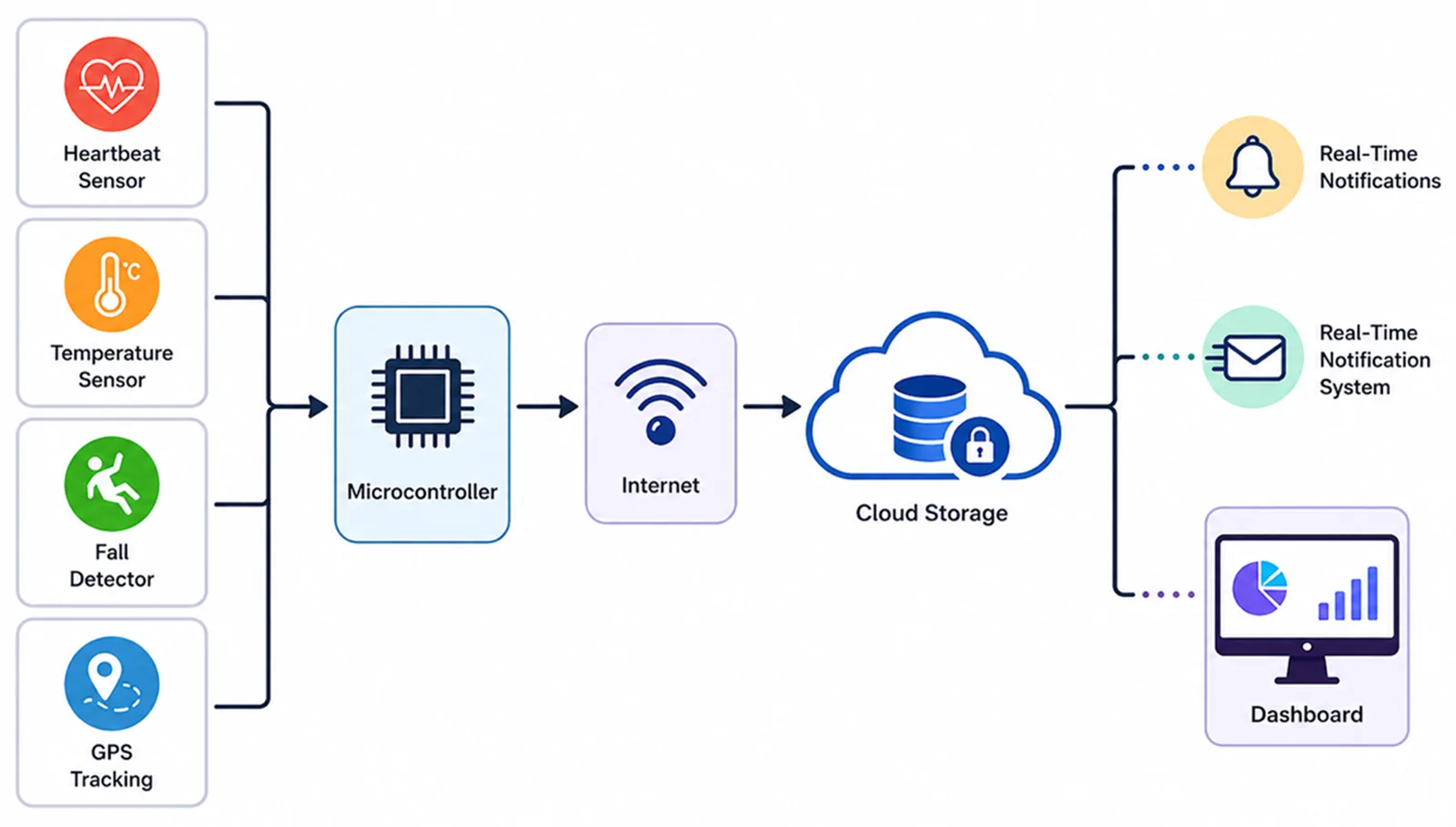
Block diagram of system components.

**Fig 3:**
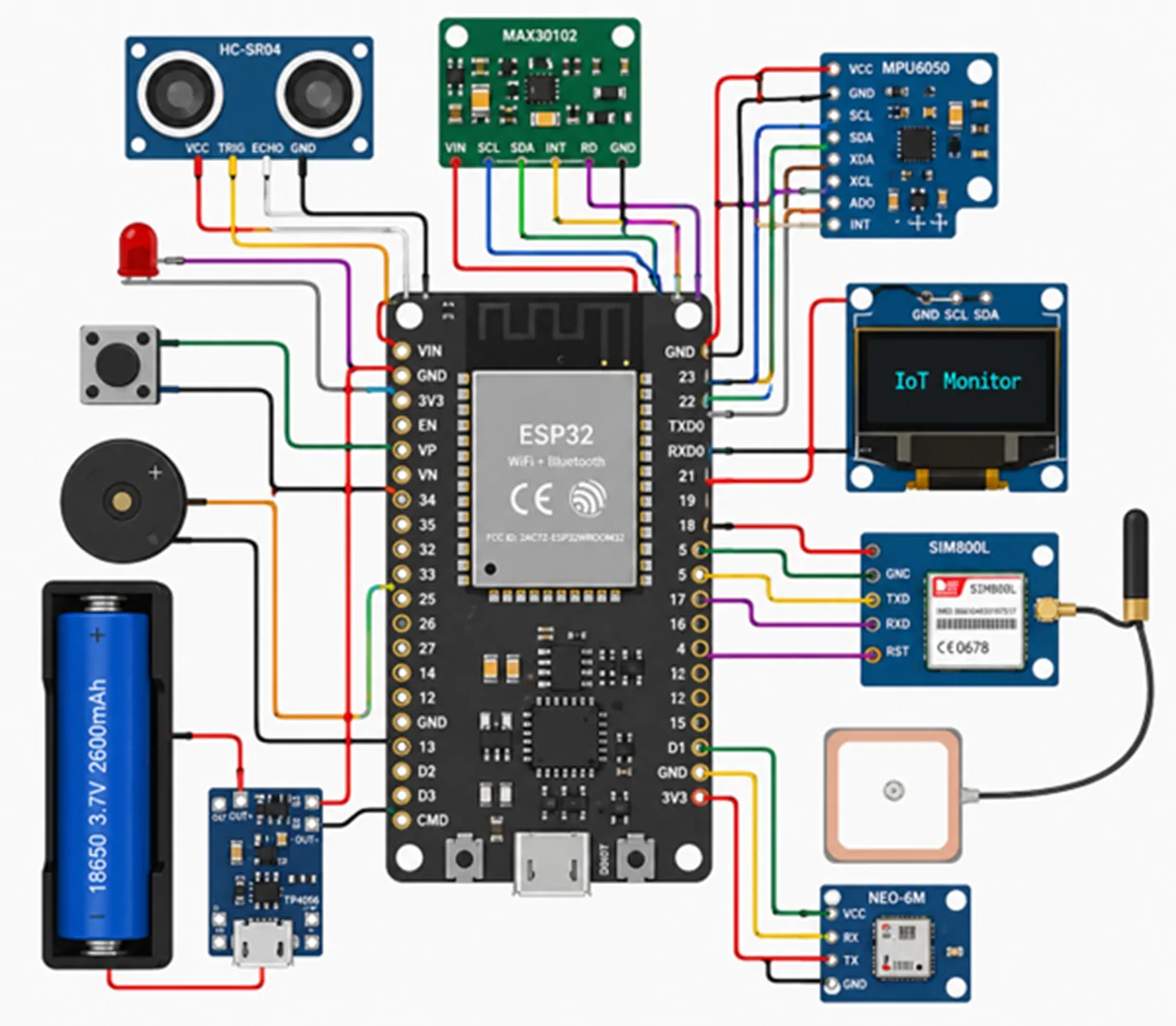
Simulation circuit.

**Fig 4:**
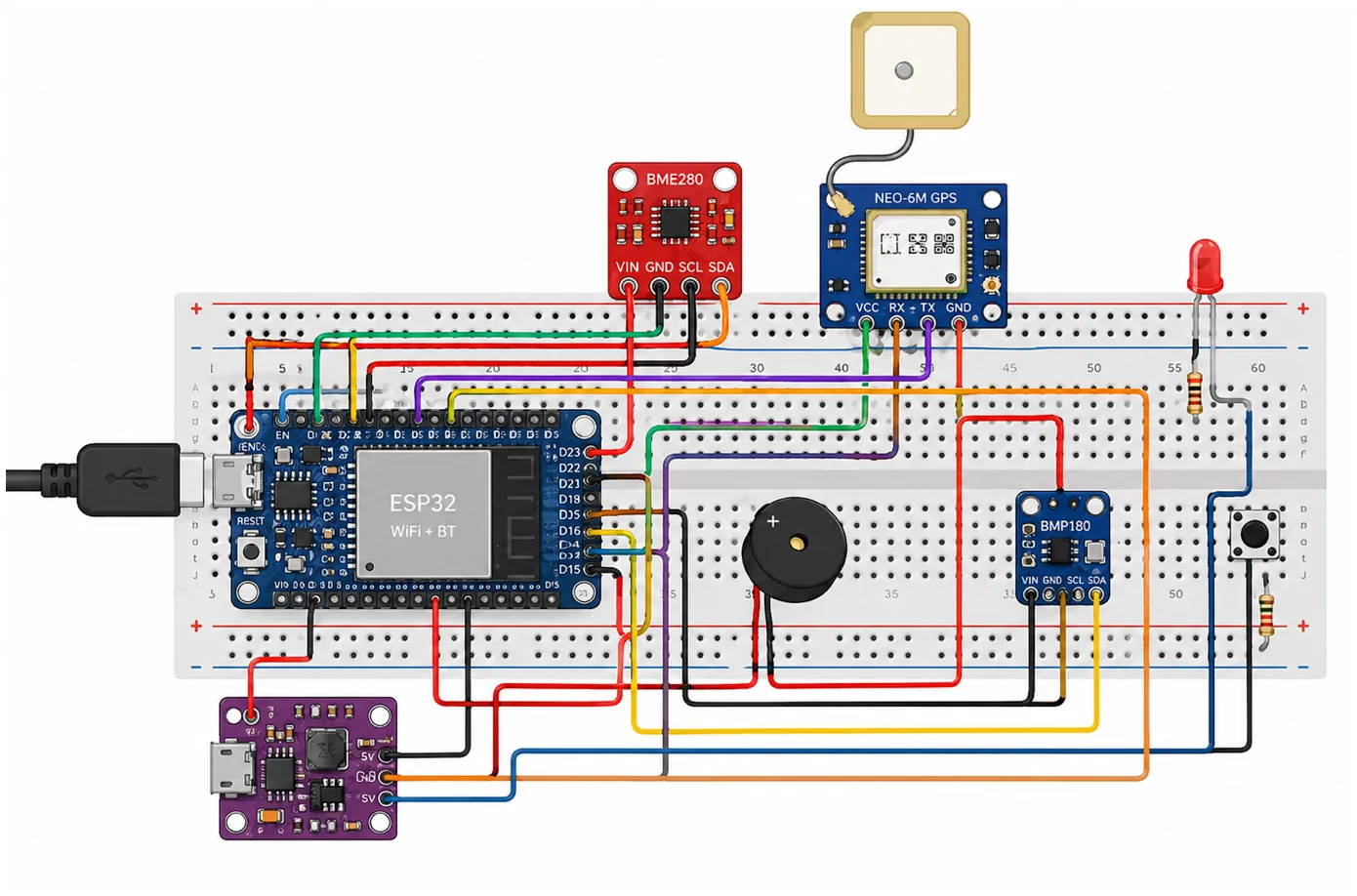
Prototype circuit implementation.

The main hardware of the proposed SSPS is a simple wearable device that relies on low power. It works via an internet microcontroller named “ESP32.” This component provides connectivity via both Wi-Fi and Bluetooth. This design focuses on facilitating convenient data transmission to the server, which is “Firebase,” and to the mobile application. Many sensors were added to ensure accurate monitoring of a Monitored Individual, such as an infrared sensor called “MLX90614” for measuring contactless body temperature, another sensor for monitoring both pulse oximetry and heart rate called “MAX30100,” and a sensor called “MPU6050” for detecting sudden movements or falls. Additionally, a GPS module called “A9G” is used to support accurate geolocation and continuous real-time tracking. Furthermore, the device includes an LED indicator and a buzzer for providing audible alerts when an SOS signal is triggered. Fig 2 illustrates the block diagram of signal flow from sensors to the mobile application, while Fig 3 displays the circuit of the proposed smart system, and Fig 4 displays how the circuit looks at the implementation stage.

### Software development

**Fig 5:**
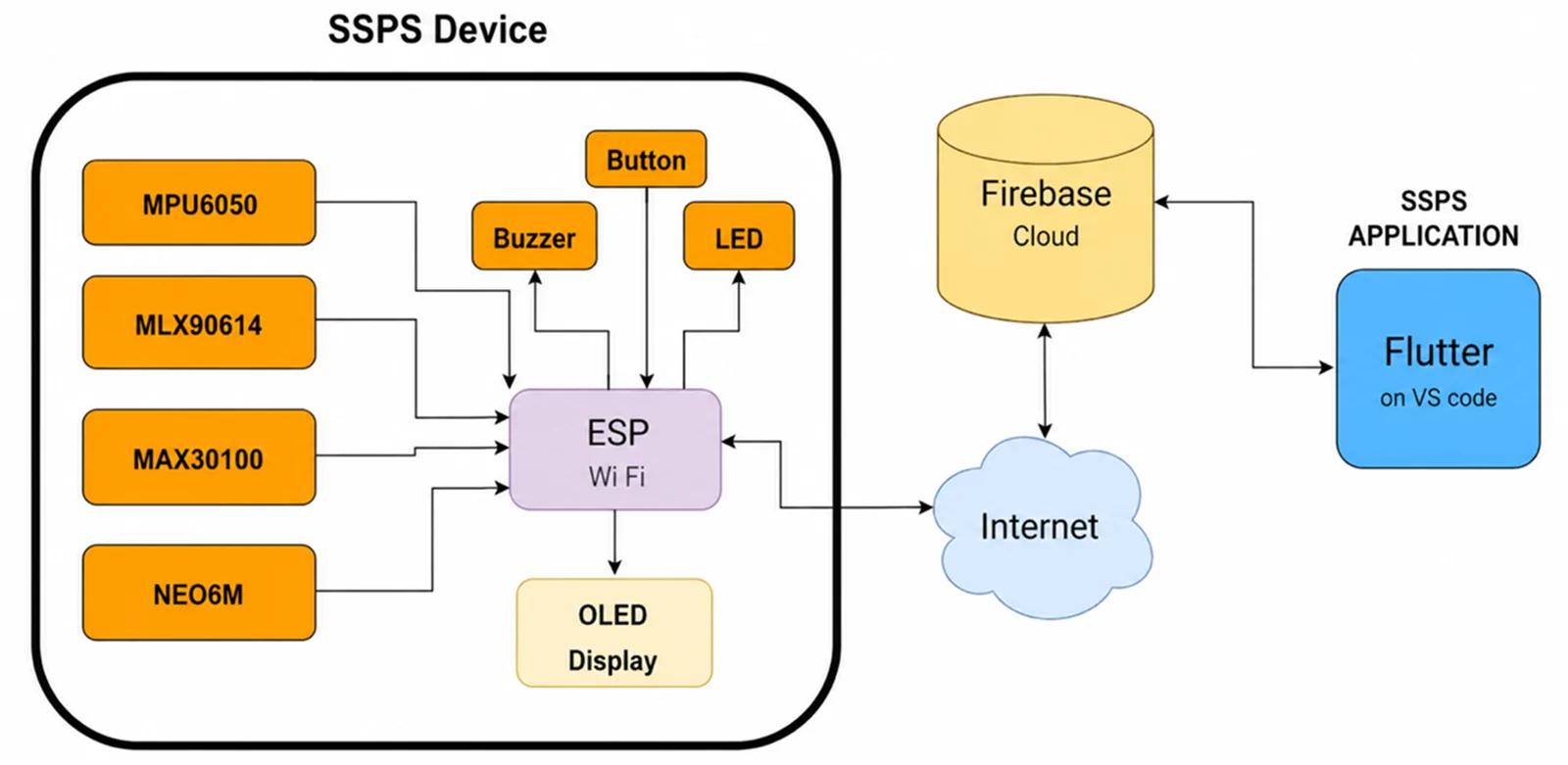
System input/output block representation.

As for the SSPS software, it includes a mobile app for Caregivers and a real-time Firebase (https://firebase.google.com/docs/database) for monitoring. The C++ language was used with the Arduino (https://www.arduino.cc) framework. The mobile app was built using Flutter (https://flutter.dev). Fig 5 shows the system’s input and output, which explain how sensor data translates into specific actions, such as alerts and dashboard updates.

**Fig 6:**
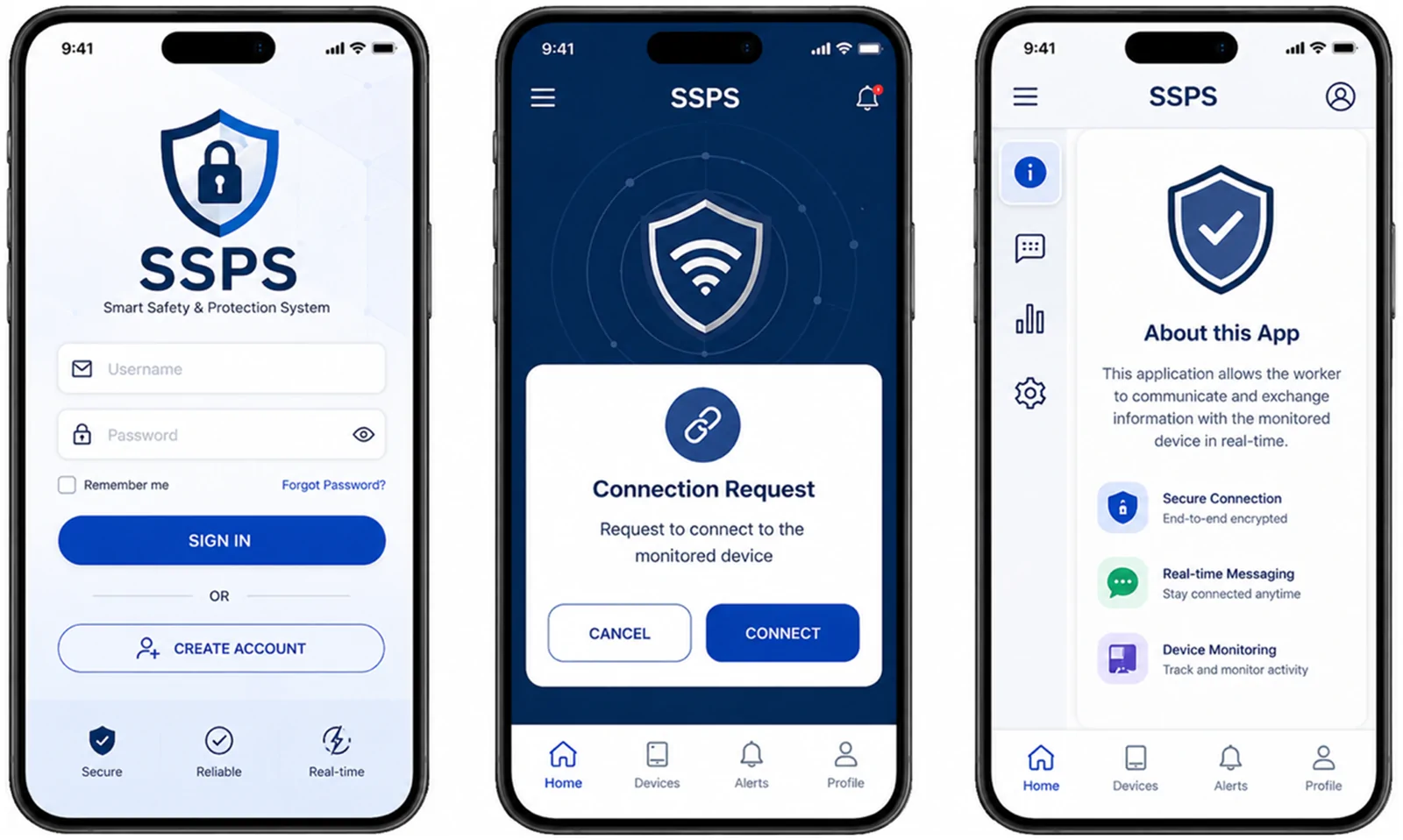
Mobile application interfaces.

**Fig 7:**
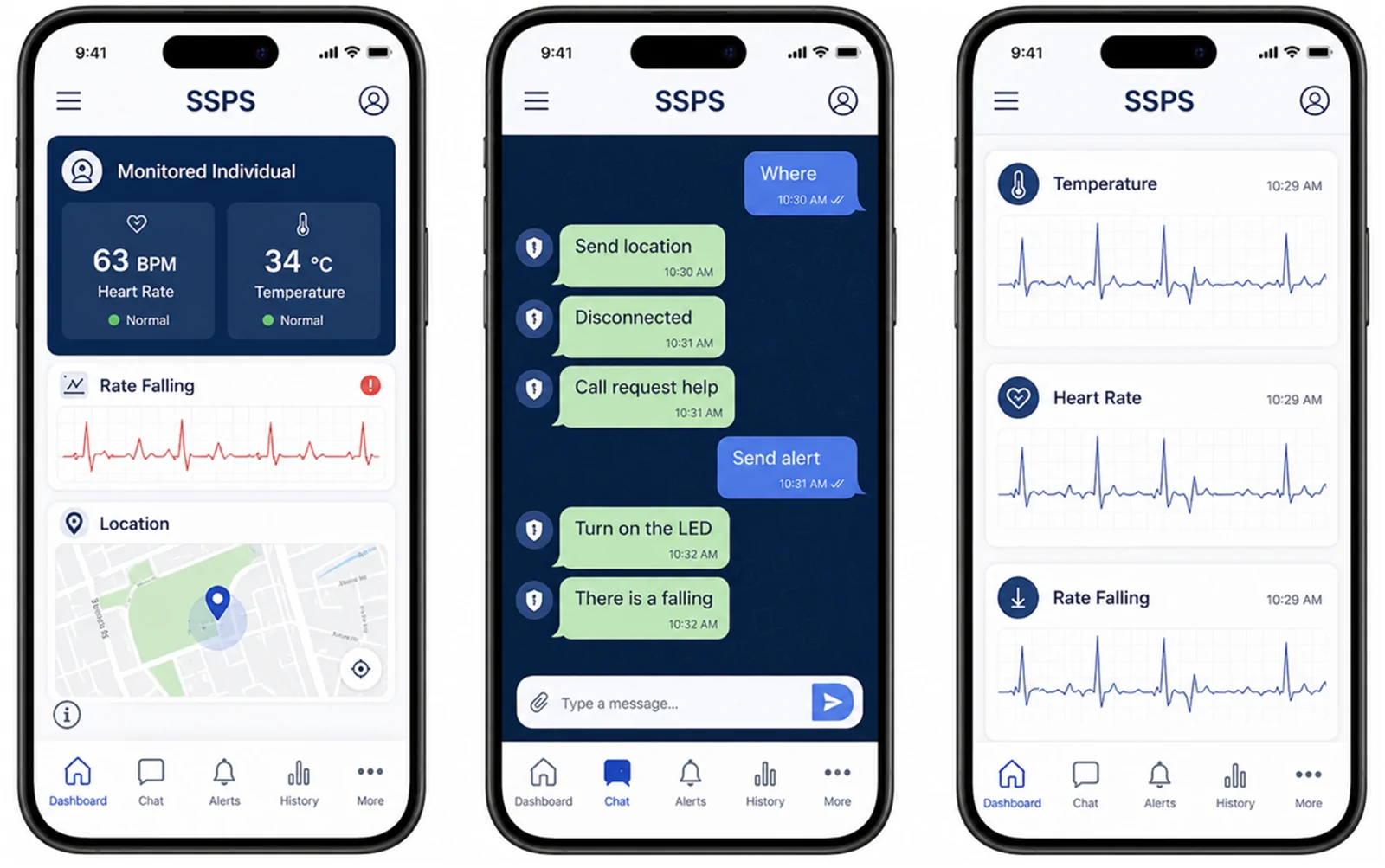
Sensor data display in the mobile app.

Seven primary screens were designed for the mobile application. This was done to provide Caregivers with complete and intuitive control of the SSPS. Firstly, a sign-up page for Caregiver account registration, which leads to a secure login page for Firebase authentication. The dashboard displays various metrics, including temperature and heart rate. Furthermore, there is a chat interface to enable interactive communication with the device to retrieve the required data. Finally, historical biometric data is available through a graphical sensor analysis screen with clear, visual charts, allowing Caregivers to track everything to ensure the Monitored Individual’s safety at all times. Figs 6 and 7 show the application interface and screens.

**Fig 8:**
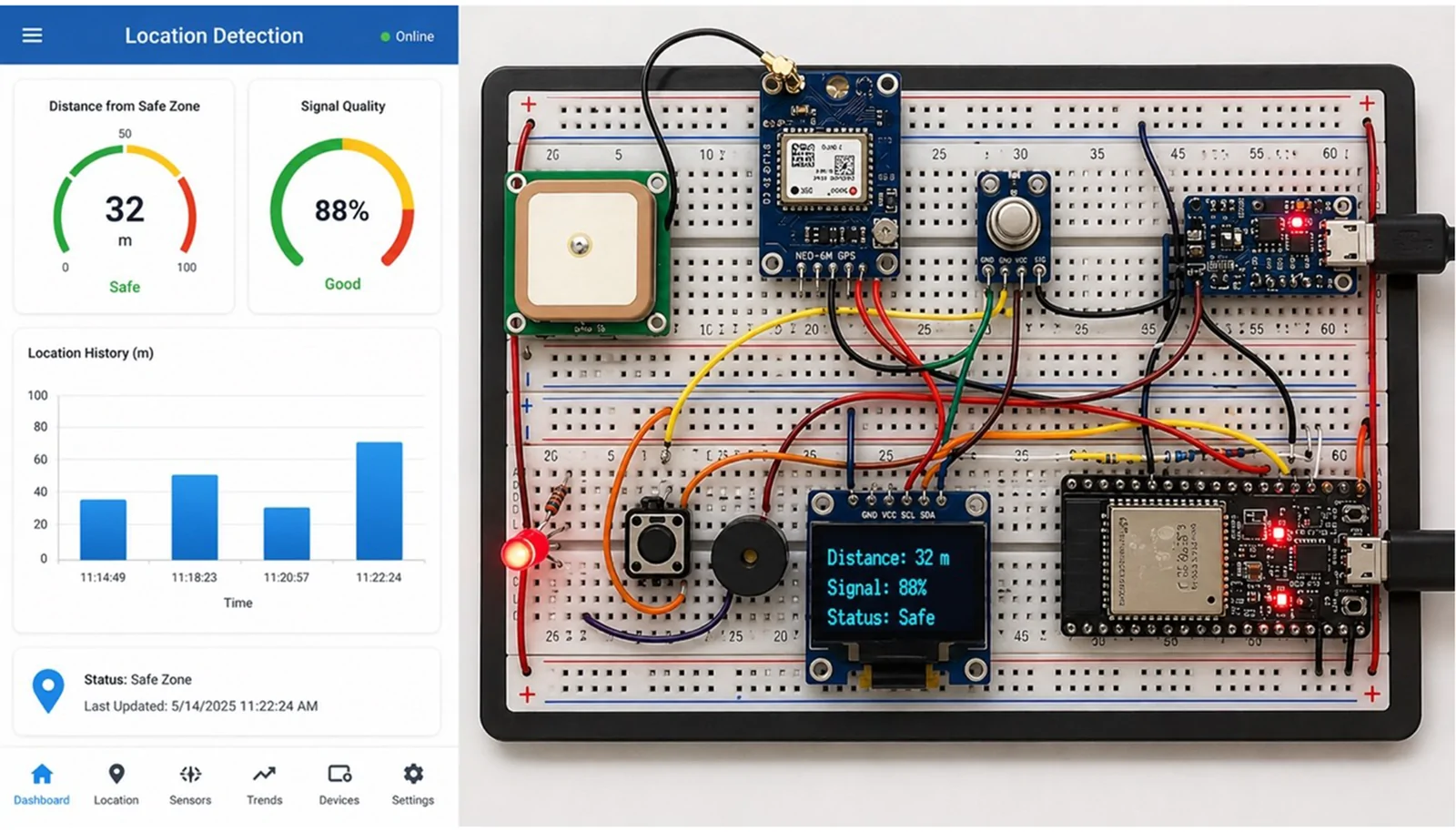
Location detection—interior view of the prototype.

Fig 8 shows the prototype of the proposed wearable device. It includes a panic button and an LED indicator. Both the panic button and the LED indicator are vital for communication in an emergency. The LED works as a remote signal that the Caregiver can activate through the application. On the other hand, the panic button sends an immediate distress message along with the GPS location to the server, and to the Caregiver’s mobile application, when pressed.

A structured engineering methodology guided our development of the proposed SSPS, involving hardware-software integration, data pipeline configuration, and testing under real-world scenarios. Each sensor in the proposed SSPS went through a calibration phase to ensure accurate and reliable measurements. The MLX90614 infrared sensor was verified against standard thermometers across multiple readings to correct for ambient temperature drift.

The system enters the Monitored Individual’s position coordinates into the database once every minute for location detection. An “Out of Perimeter” notification appears on the onboard LCD screen and the new coordinates are presented on the dashboard of the mobile application if the Monitored Individual leaves the specified boundary. The LED and buzzer are both turned on for 5 seconds to indicate the event, and a push notification is also sent to the Caregiver to warn them.

**Algorithm 1: GPS location detection algorithm**



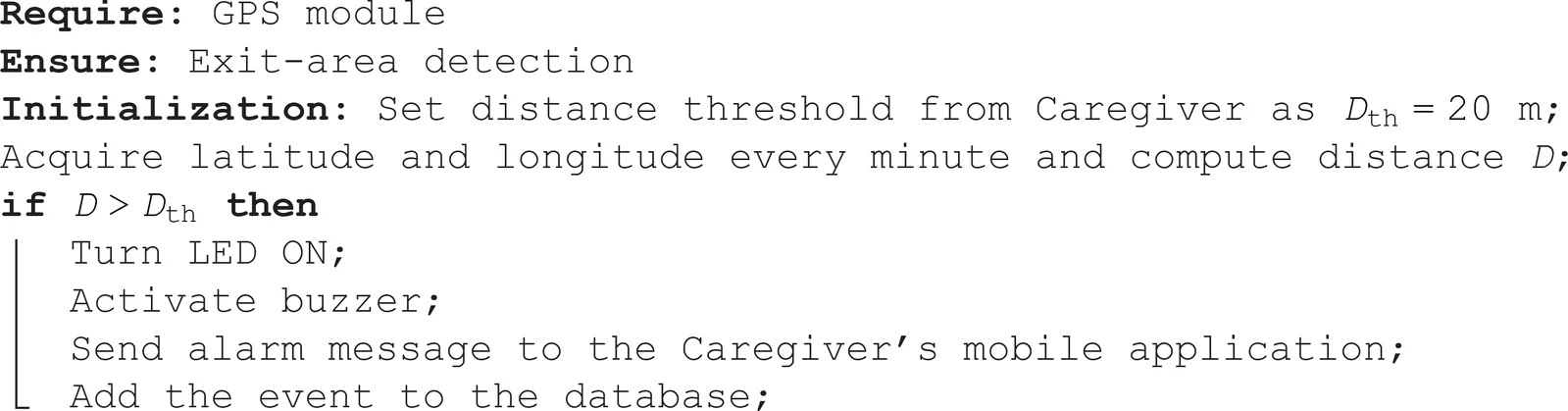



**Fig 9:**
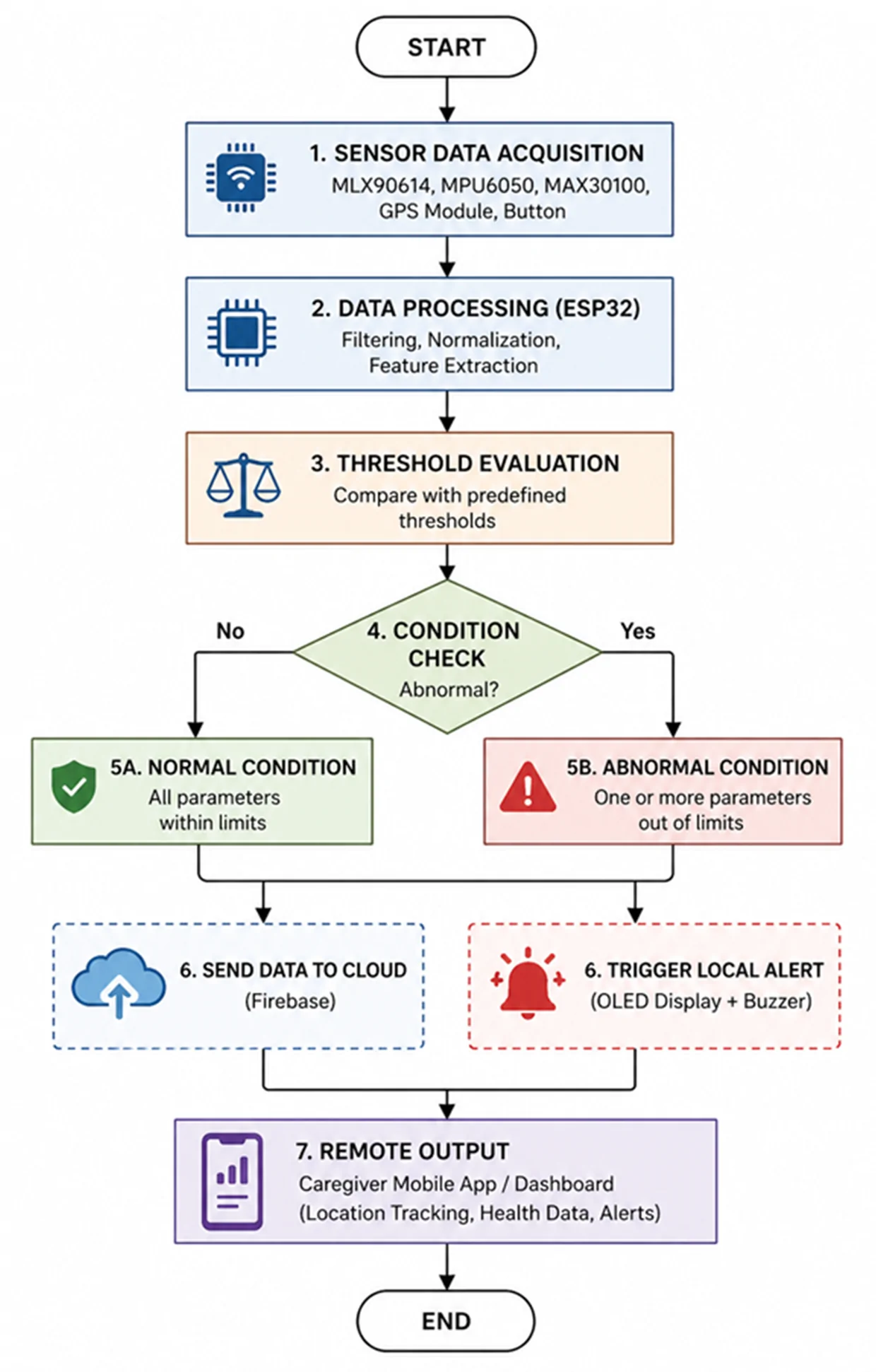
Integration testing project overview.

As a visual representation of the integration and testing of the system, Fig 9 presents an overview of the project based on Cloud Firestore (https://firebase.google.com/docs/firestore), which stores data as a collection of documents. Each document contains its own set of data fields, or subsets, which organize information in a structure similar to rows and tables for better compatibility. It supports various data formats, including objects, text strings, numbers, and Boolean values. Additionally, Cloud Firestore can store timestamps, geographic locations, and references, making it easy to manage vital information about the Monitored Individual, monitor their environment, and take timely action to ensure their safety from potential dangers.

### Deep Learning Integration

**Fig 10:**
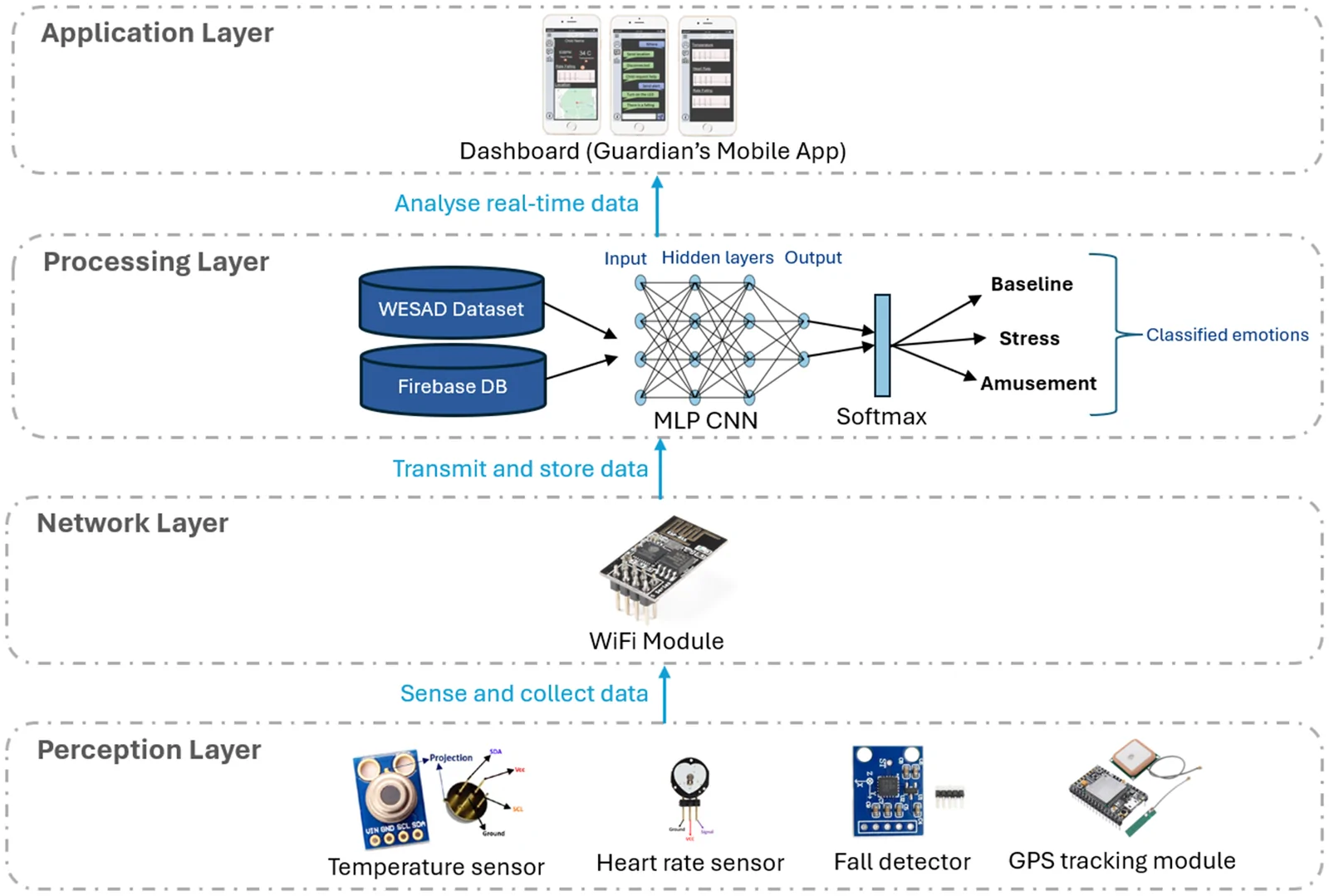
Layered overall architecture of the proposed system.

To enhance SSPS capabilities, we incorporated a deep learning component to classify a Monitored Individual’s emotional and physiological states based on wearable-sensor data. The wearable-sensor data collected from the proposed bracelet is fed into a deep learning model to predict the emotional or physiological state of the Monitored Individual and send the results to the Caregiver’s mobile application. To train the deep learning model and achieve high accuracy, we needed a large dataset. Thus, we utilized the WEarable Stress and Affect Detection (“WESAD”) dataset (https://www.kaggle.com/datasets/orvile/wesad-wearable-stress-affect-detection-dataset). According to our goal, the movement factor is essential: if the Monitored Individual’s temperature and heart rate are high while they are moving, this is considered normal or baseline, but if they are high while the Monitored Individual is not moving, it is an indication of fright or stress. Therefore, we benefit from the movement data provided by the WESAD dataset, in addition to the recorded physiological signals. Fig 10 illustrates the layered overall architecture of the proposed model integrated with deep learning technology.

All sensing devices are in the perception layer, as presented in Fig 10. The perception layer sends the collected physiological data through the network layer to the processing layer. The deep learning model, along with the database and dataset, is located within the processing layer. The classified emotions generated from this layer are sent to the dashboard mobile app located in the application layer.

#### WESAD dataset.

WESAD is a freely available dataset that is widely used for stress detection, emotion recognition, and wearable sensor validation. Two sensor devices were used simultaneously to collect the WESAD dataset: RespiBAN Professional (chest-worn) and Empatica E4 (wrist-worn). The dataset includes multimodal sensor data/signals as illustrated in Table 3.

**Table 3:** Explanation of WESAD dataset signals.

Signal	Description	Units	Typical Use
ACC	Three-axis accelerometer	*g* (acceleration due to gravity)	Detection of chest, hand, or wrist movement, posture, and motion artifacts
ECG	Electrocardiogram	millivolts (mV)	Heart rate, heart rate variability (HRV), and R-peak detection
BVP	Blood volume pulse (from PPG sensor)	arbitrary units (optical intensity)	Heart rate and HRV estimation
TEMP	Body temperature	°C	Skin temperature monitoring and stress/calm state indication
RESP	Respiration	relative amplitude	Breathing rate, rhythm, and stress-related changes
EMG	Electromyogram	millivolts (mV)	Chest muscle activity
EDA	Electrodermal activity	microsiemens (μS)	Skin conductance and stress indication

Each of the signals presented in Table 3 has 700 samples per second, providing very fine temporal resolution. There are fifteen subject datasets in the WESAD repository; we trained our deep learning model on one of them as a sample (S16, comprising 112 total physiological measurements). The generated labels from the employed signals are classified into three main classes, as shown in Table 4.

**Table 4:** Ground-truth labels indicating emotional states.

Label	Emotion State	Description
0	Baseline	Neutral or calm state
1	Amusement	Positive emotional state
2	Stress	Negative emotional state

These classes represent the output of our deep learning model, which is transmitted to the linked mobile application as an accurate, real-time analysis of the emotional state.

The data were collected under three different situations. First, participants were sitting quietly, relaxed, and breathing normally. Second, they performed the Trier Social Stress Test (TSST), involving arithmetic and public speaking tasks under time pressure. The third situation induced amusement by having participants watch funny video clips to induce happiness. We note that the WESAD dataset consists of adult participants collected under controlled experimental settings. We selected it due to its high-quality, multimodal physiological recordings, including ECG signals, which are widely used in stress and emotion recognition research. It provides well-annotated data, making it suitable for initial model development and benchmarking.

#### Processing.

**Fig 11:**
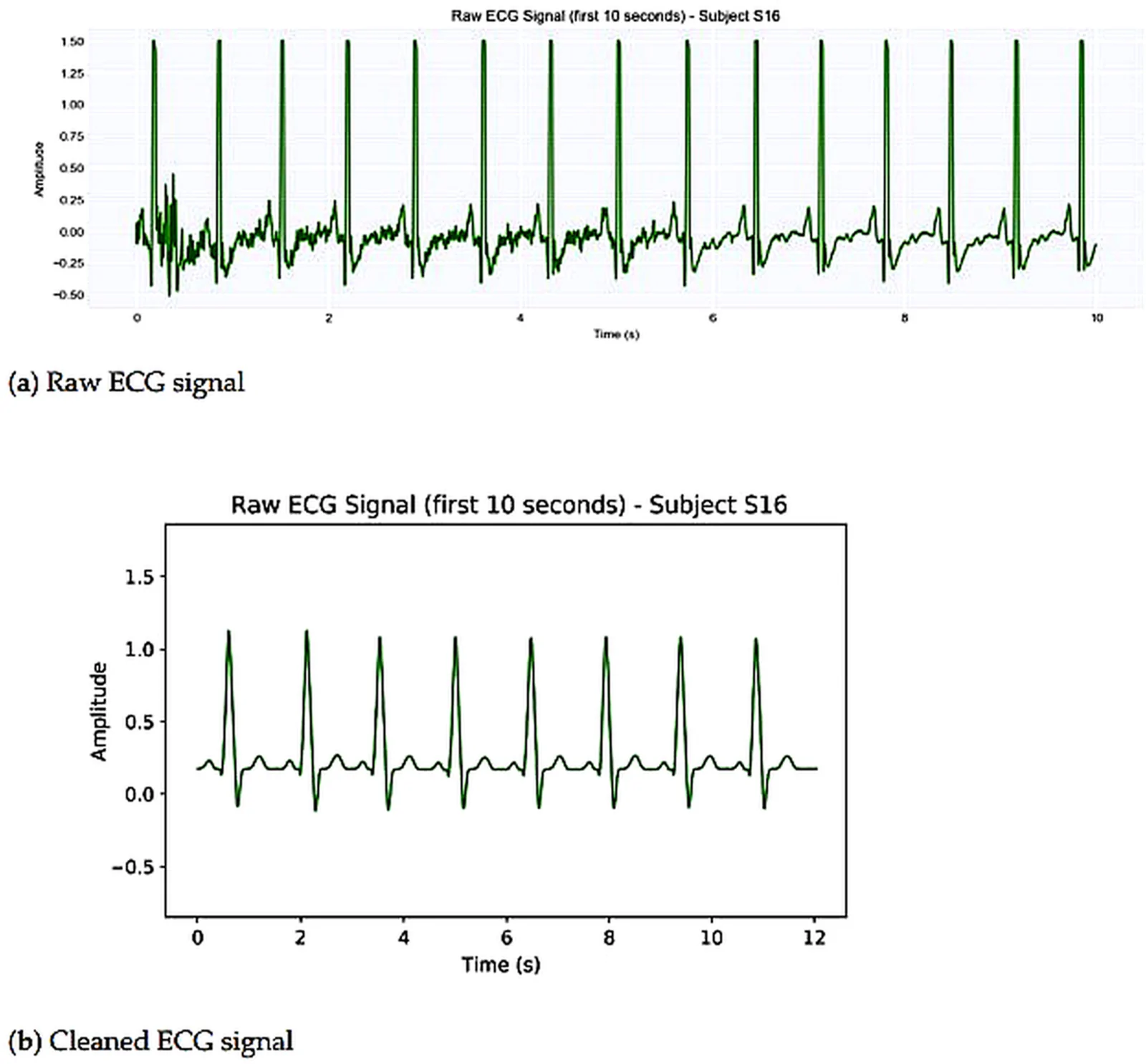
Comparison of raw and cleaned ECG signals for Subject S16 over the first 10s. (A) Raw ECG signal. (B) Cleaned ECG signal.

After importing the required libraries and loading the dataset, we pre-processed the data as illustrated in Figs 11 to Fig 13.

**Fig 12:**
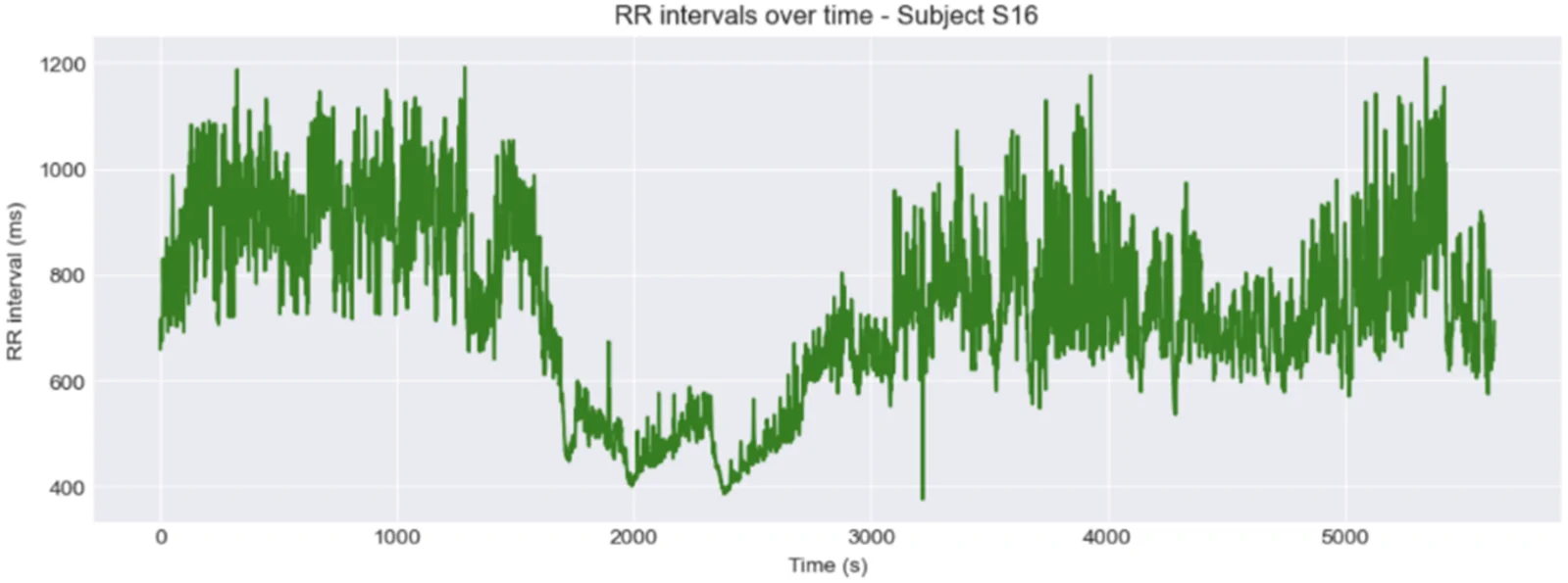
RR intervals over time.

**Fig 13:**
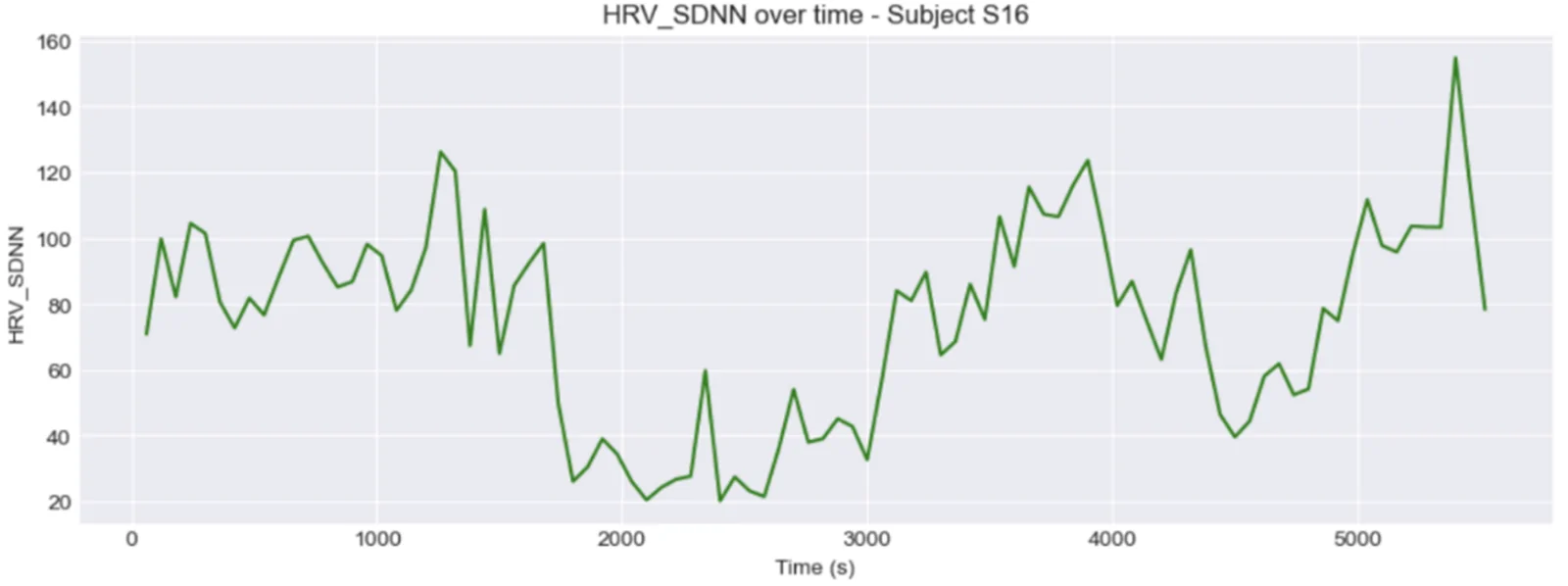
HRV_SDNN over time.

Fig 11 displays 10 seconds of ECG signal for a sample subject (S16). The x-axis shows time in seconds, while the y-axis shows the signal amplitude. This visualization helps illustrate how clean or noisy the original ECG waveform is. As we notice in Fig 11a, the QRS peaks (sharp upward spikes that represent heartbeats) are visible, but the baseline is not perfectly flat, and the smaller components (P and T waves) are often distorted. However, in Fig 11b, we applied NeuroKit2’s filtering technique, which removed drift and noise and cleaned the signal as a preparation step for training the model.

After detecting R-peaks (the sharp ECG spikes for each heartbeat), the script computes RR intervals (the time difference between consecutive heartbeats). The plot in Fig 12 shows heartbeat-to-heartbeat variation, which is used later for HRV features.

Fig 13 plots how SDNN changes during the experiment, helping visualize physiological responses to stress or amusement. SDNN (Standard Deviation of NN intervals) is a key HRV feature reflecting heart rate variability.

#### Deep learning model compilation.

Machine Learning (ML) is a branch of artificial intelligence that enables systems to learn patterns and relationships from data to make predictions or decisions without explicit programming. It relies on algorithms that improve their performance through experience as they are exposed to more data. Deep Learning (DL), on the other hand, is a specialized subset of machine learning that uses artificial neural networks with multiple layers to model complex, non-linear relationships in large-scale datasets. By automatically extracting high-level features from raw data, deep learning techniques achieve superior performance in tasks such as image recognition, speech processing, and physiological signal analysis.

To classify emotions, we used a Multi-Layer Perceptron (MLP), a fundamental type of neural network in DL. Deep learning models use multiple layers of interconnected neurons to learn complex patterns from data, and the MLP is one of the simplest forms of such architectures. Specifically, an MLP consists of an input layer, one or more hidden layers, and an output layer, where each neuron is fully connected to the next layer. When an MLP has multiple hidden layers, it becomes a deep neural network and a core building block of deep learning. Therefore, the MLP can be viewed as both a foundational model within DL and a stepping stone toward more advanced architectures such as Convolutional Neural Networks (CNNs) and Recurrent Neural Networks (RNNs).

The MLP was trained using forward propagation, in which the input data passes through the network layer by layer, and each neuron computes its output based on its weights, inputs, and biases. The model then calculated the loss by comparing its output with the target output, and the loss function measured the prediction error. Lastly, backpropagation was performed, sending the error backward through the network and updating weights to minimize the loss. This process is repeated over multiple epochs to improve accuracy.

Regarding the learning parameters, we used a learning rate α=0.1to control how much the model’s weights are adjusted during training. To train the MLP deep learning model, we tried both the Stochastic Gradient Descent (SGD) optimization algorithm and the Adam optimizer (batch size = 64). Moreover, to enable the network to learn complex relationships within the physiological measurements, extract features from the data, and ultimately predict the emotion as one of three classes, we tested both Softmax and Sigmoid activation functions. Lastly, to gradually improve accuracy, the learning process was repeated for 2000 epochs. Full code is available on GitHub (https://github.com/ManalKhayyat/smart_bracelet/blob/main/smart_bracelet.ipynb).

## Experimental evaluation and results

We began our experiment by importing the necessary Python libraries for deep learning, first employing “NeuroKit2,” an open-source library designed for neurophysiological signal processing and biopsychological data analysis. We then utilized the “torch.nn” submodule from PyTorch, a popular deep learning library that contains classes and functions for building and training neural networks. Lastly, we used “sklearn,” a machine learning utility that provides simple and efficient tools for data analysis, preprocessing, and modeling.

The dataset was divided into training and testing sets using a 70:30 split, ensuring that samples from each class (baseline, stress, and amusement) were proportionally represented. Moreover, we addressed the issue of class imbalance and ensured fair model training by applying stratified sampling during the training process.

### Results and discussion

**Fig 14:**
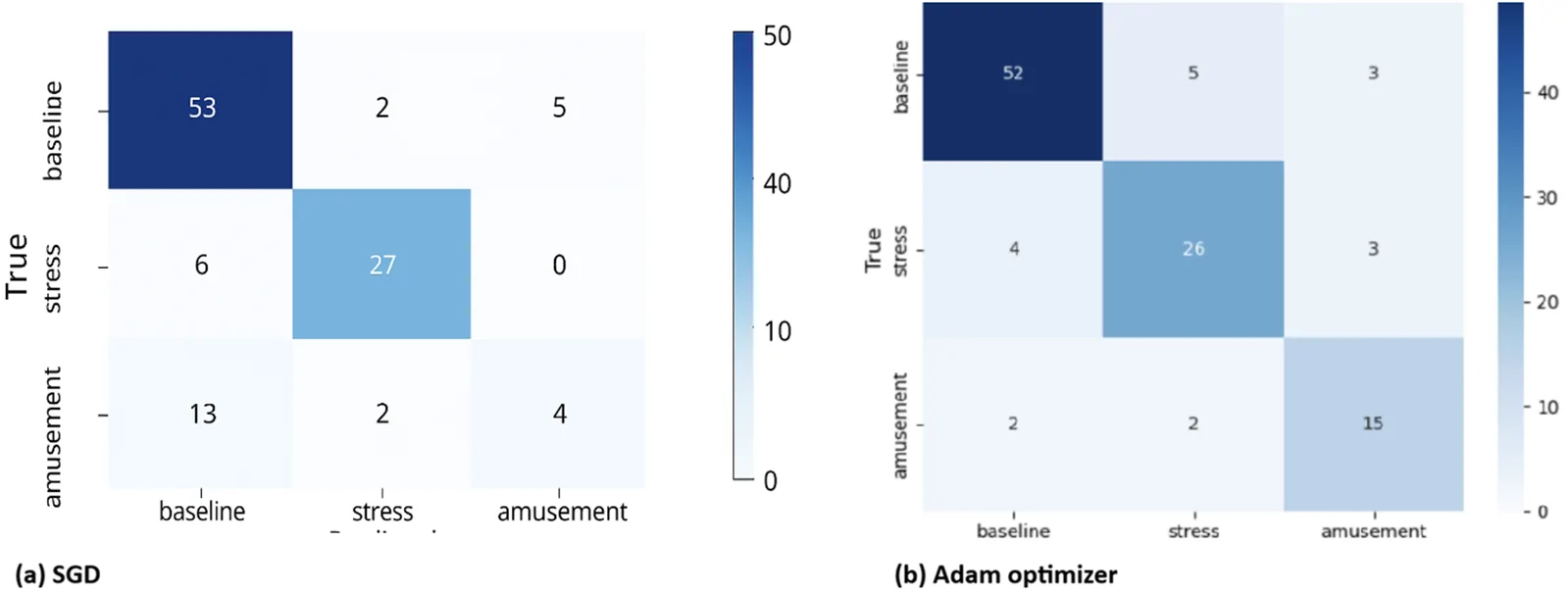
Generated confusion matrices using SGD and Adam optimizer.

To evaluate the developed MLP deep learning model, we generated the confusion matrices illustrated in Fig 14.

The confusion matrices in Fig 14 assist in comparing the classified emotions with the ground-truth ones, since the rows contain the true emotions while the columns contain the classified emotions. We notice a clear diagonal forming inside the confusion matrices, containing the largest numbers, which indicates that the model was able to successfully classify the emotions. However, Fig 14a used the SGD optimizer, and we can notice that the model struggles with classifying the “amusement” class, since 13 out of 19 samples were confused with baseline and only 4 samples were predicted correctly. In contrast, the model performed well with the other two classes (baseline and stress). The model performs quite well for baseline, since most measurements were classified correctly (53 samples correctly classified as baseline, 2 samples wrongly predicted as stress, and 5 samples wrongly predicted as amusement). The model also shows good performance for the stress class (27 samples correctly identified as stress, 6 samples mislabeled as baseline, and no samples mislabeled as amusement).

To calculate the overall model’s accuracy, we divided the total correct predictions by the total number of samples and reached 0.75 accuracy.


$$\begin{array}{c} \text{Accuracy}=\frac{84}{112}\approx 75\% \end{array}$$

Moreover, we noticed that accuracy improved in Fig 14b, which used the Adam optimizer instead of SGD. The accuracy reached 0.83, and the diagonal became clearer, since fifteen samples from the amusement class were predicted correctly.

Lastly, we computed the precision, recall, and F1-score for each class, as summarized in Table 5.


$$\begin{array}{c} \text{Precision}=\frac{TP}{TP+FP} \end{array}$$ (1)

$$\begin{array}{c} \text{Recall}=\frac{TP}{TP+FN} \end{array}$$ (2)

$$\begin{array}{c} \text{F1-score}=2\times \frac{\text{Precision}\times \text{Recall}}{\text{Precision}+\text{Recall}} \end{array}$$ (3)

**Table 5:** Evaluation metrics using SGD and Adam optimization algorithm.

	SGD		
	Baseline	Stress	Amusement
**Precision**	0.736	0.871	0.444
**Recall**	0.883	0.818	0.211
**F1-Score**	0.803	0.844	0.285
	**Adam**		
**Precision**	0.897	0.788	0.714
**Recall**	0.867	0.788	0.790
**F1-Score**	0.881	0.788	0.759

Where *TP* represents True Positives (correct predictions for that class), *FP* represents False Positives (other classes predicted as this class), and *FN* represents False Negatives (this class predicted as other classes).

**Fig 15:**
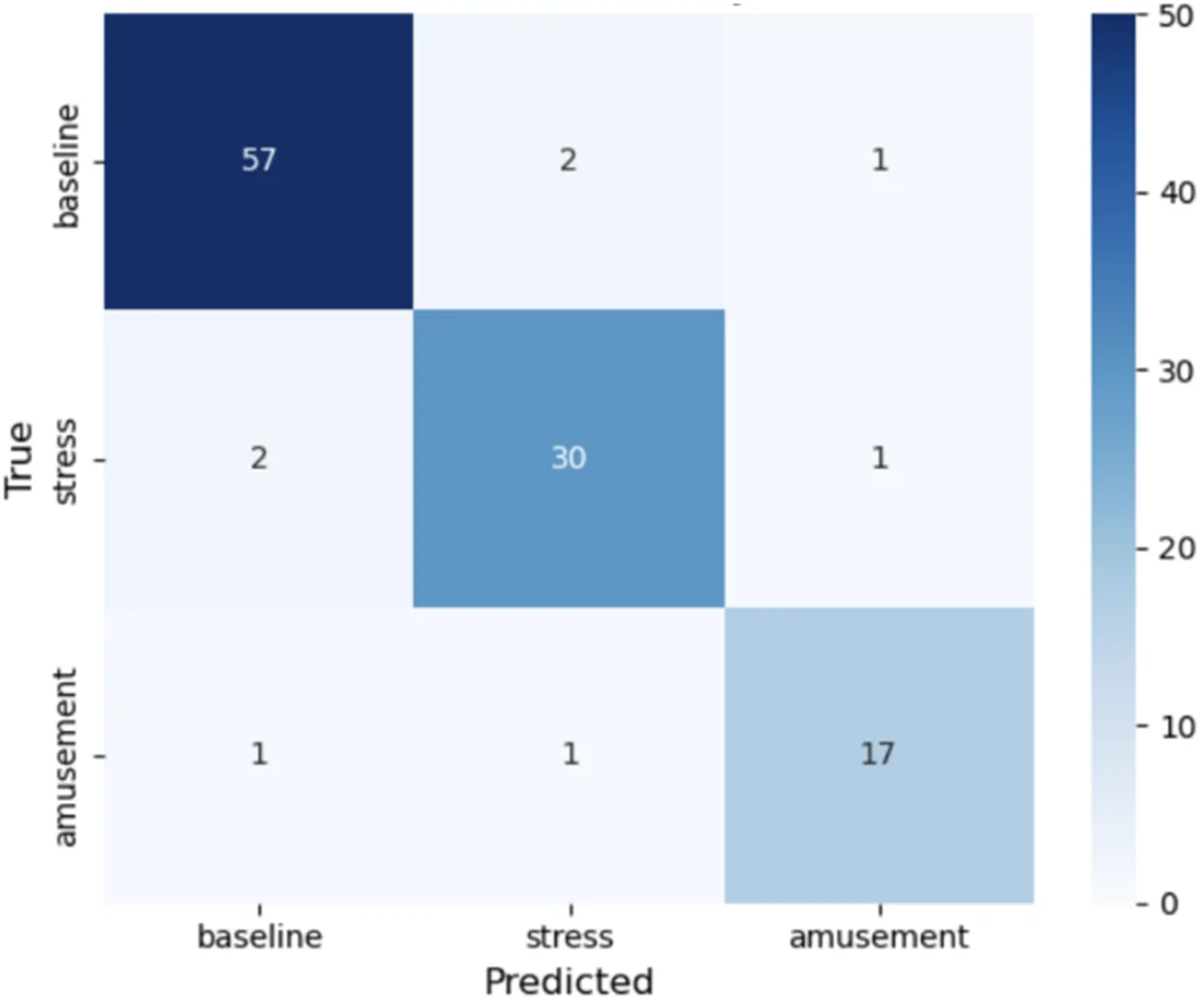
Generated confusion matrix using Adam optimizer and Softmax function.

From Table 5, we conclude that the Adam optimization algorithm works better for our problem than SGD. We therefore used this algorithm in our deep learning model. To further improve accuracy, we repeated the experiment using Softmax instead of Sigmoid as the activation function. With this new experiment, we reached 0.93 accuracy. The new confusion matrix is presented in Fig 15.

As shown in Fig 15, the diagonal is bold and clear. Only 3 samples were mislabeled in the baseline class, while 57 samples were predicted correctly. Similarly, only 3 samples were mislabeled in the stress class, while 30 samples were predicted correctly. The amusement class performs best, with only 2 samples mislabeled and 17 predicted correctly.

The newly computed evaluation metrics (precision, recall, and F1-score) are 0.950 for the baseline class, 0.909 for the stress class, and 0.895 for the amusement class, respectively. Table 6 summarizes the detailed per-class classification performance, while Table 7 presents the overall performance metrics.

**Table 6:** Per-class classification performance metrics.

Class	Precision	Recall	F1-Score
Baseline	0.950	0.950	0.950
Stress	0.909	0.909	0.909
Amusement	0.895	0.895	0.895

**Table 7:** Overall metrics.

Metric	Value
Accuracy	0.93
Macro Precision	0.92
Macro Recall	0.92
Macro F1-Score	0.92

The MLP deep learning model shows substantial improvement over previous experiments, as shown by its higher prediction accuracy. Therefore, we adopted these optimized learning configurations for subsequent analyses.

In summary, employing the Adam optimizer with a batch size of 64 improved model accuracy from 75% to 83%. Furthermore, integrating the Softmax activation function enhanced accuracy to 93%, demonstrating a significant advancement in the model’s ability to accurately distinguish Monitored Individuals’ emotional states under optimized learning conditions. The combination of wearable sensing technology and deep learning techniques has proven effective for developing a robust framework capable of achieving optimized real-time emotion monitoring.

**Fig 16:**
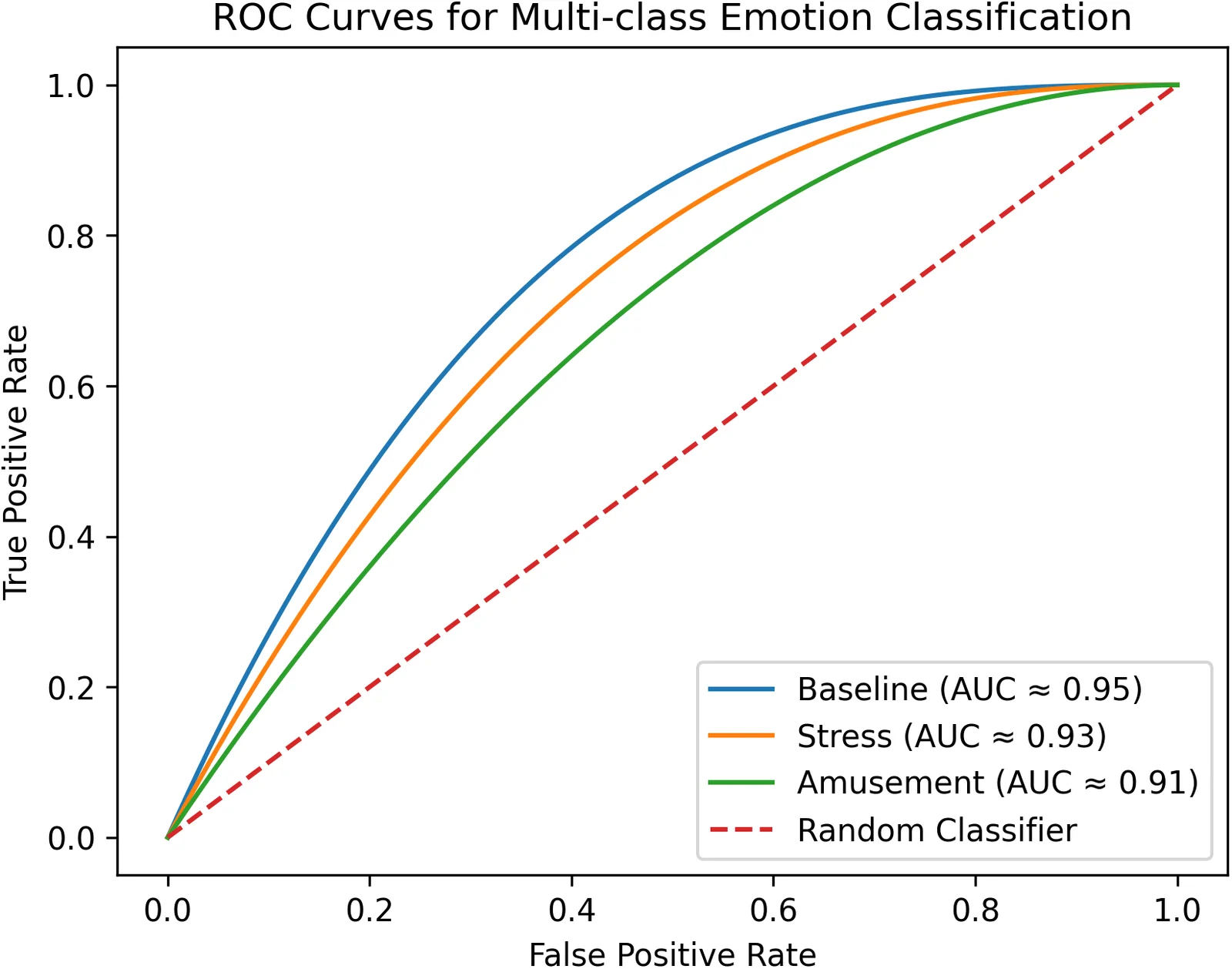
ROC curves for multi-class emotion classification. The receiver operating characteristic (ROC) curves illustrate the classification performance of the proposed model for the Baseline, Stress, and Amusement classes. The model achieved area under the curve (AUC) values of approximately 0.95, 0.93, and 0.91, respectively, demonstrating strong discriminative capability across all emotion categories. The dashed diagonal line represents the performance of a random classifier.

The ROC curves further demonstrate the effectiveness of the proposed multi-class emotion classification model. As shown in Fig 16, the model achieved high discriminative performance across all emotion classes, with AUC values of approximately 0.95, 0.93, and 0.91 for the Baseline, Stress, and Amusement classes, respectively (Tables 6 and 7).

These results confirm that the proposed deep learning model achieves robust and reliable performance in multi-class emotion recognition, with balanced accuracy across all classes and significant improvement over previous configurations.

To further validate the effectiveness of the proposed deep learning approach, a comparison with traditional non-ML/DL methods is essential. Conventional systems for emotion or stress detection typically rely on rule-based or threshold-based analysis of physiological signals such as heart rate and ECG features. However, such approaches are limited in their ability to capture complex, non-linear relationships within biosignals. Previous studies have reported that traditional machine learning or threshold-based methods generally achieve accuracies in the range of 60–75% for emotion recognition tasks using physiological data, particularly when distinguishing between closely related emotional states (e.g., baseline and amusement) [23,24].

In contrast, the proposed MLP-based deep learning model achieved an overall accuracy of approximately 93% (104 correct predictions out of 112 samples), as demonstrated in Fig 15. This represents an improvement of approximately 18–33% over traditional approaches. In addition, the confusion matrix shows significantly reduced misclassification across all classes, with only 8 errors out of 112 samples, indicating enhanced robustness and generalization capacity. The superior performance of the deep learning model can be attributed to its ability to automatically learn hierarchical feature representations from ECG signals, which are difficult to model using conventional techniques.

These findings are consistent with recent literature emphasizing that deep learning models outperform traditional methods in physiological signal analysis due to their capacity for feature learning and non-linear modeling [25,26]. Therefore, the incorporation of DL into the proposed system substantially improves its accuracy, reliability, and suitability for real-time emotion recognition applications.

## Conclusion

This work presented the design and implementation of the SSPS, which combines wearable sensors, mobile connectivity, and intelligent analysis to support Monitored Individuals’ safety. The system demonstrated its ability to monitor vital signs, detect falls, track location, and issue immediate alerts when unusual conditions occur. This was achieved through the development of a compact IoT-based bracelet and a user-friendly mobile application. The integration of a deep learning model further strengthened the SSPS by enabling accurate emotion recognition from wearable-sensor data. The optimized MLP network achieved 93% accuracy, offering timely and meaningful insight into Monitored Individuals’ stress levels and overall well-being.

What makes this work meaningful is not only the technical outcome but also the practical impact it aims to create. The system was intentionally built with families in mind: simple enough for everyday use, yet robust enough to provide real-time support during emergencies. While the prototype successfully passed functional tests and showed encouraging results, especially with the optimized deep learning model, it also highlighted areas where continuous refinement can lead to a more mature and reliable solution.

Several directions can help strengthen and expand the SSPS for future work. First, conducting large-scale real-world testing across age groups would allow us to validate the system in more diverse environments and refine the stress-detection model using real data rather than relying solely on the WESAD dataset. In addition, improving battery life, reducing device size, and enhancing comfort will be essential for long-term wearability. Future versions may also incorporate advanced communication modules to support areas with limited Wi-Fi access. On the software side, strengthening security and privacy features, including end-to-end encryption and more robust access controls, is necessary to protect sensitive data. Finally, adding support for caregivers, schools, and emergency services could help position the SSPS as a community-wide safety platform rather than a standalone family device.
